# Prevalence of sarcopenia in community-dwelling older adults using the updated EWGSOP2 definition according to kidney function and albuminuria: The Screening for CKD among Older People across Europe (SCOPE) study

**DOI:** 10.1186/s12877-020-01700-x

**Published:** 2020-10-02

**Authors:** Rafael Moreno-Gonzalez, Xavier Corbella, Francesco Mattace-Raso, Lisanne Tap, Cornel Sieber, Ellen Freiberger, Tomasz Kostka, Agnieszka Guligowska, Itshak Melzer, Yehudit Melzer, Axel C. Carlsson, Johan Ärnlöv, Regina Roller-Wirnsberger, Gerhard Wirnsberger, Pedro Gil, Sara Lainez Martinez, Paolo Fabbietti, Andrea Corsonello, Fabrizia Lattanzio, Francesc Formiga, on behalf of SCOPE investigators, Fabrizia Lattanzio, Andrea Corsonello, Silvia Bustacchini, Silvia Bolognini, Paola D’Ascoli, Raffaella Moresi, Giuseppina Di Stefano, Cinzia Giammarchi, Anna Rita Bonfigli, Roberta Galeazzi, Federica Lenci, Stefano Della Bella, Enrico Bordoni, Mauro Provinciali, Robertina Giacconi, Cinzia Giuli, Demetrio Postacchini, Sabrina Garasto, Annalisa Cozza, Romano Firmani, Moreno Nacciariti, Mirko Di Rosa, Paolo Fabbietti

**Affiliations:** 1 Geriatric Unit, Internal Medicine Department, Hospital Universitari de Bellvitge, Systemic Diseases and Ageing Group, Cardiovascular, Respiratory and Systemic Diseases and Cellular Aging Program, Institut d’Investigació Biomèdica de Bellvitge (IDIBELL), L’Hospitalet de Llobregat, Barcelona, Spain; 2 Hestia Chair in Integrated Health and Social Care, Faculty of Medicine and Health Sciences, Catalunya International University, Barcelona, Spain; 3 Department of Internal Medicine, Section of Geriatric Medicine, Erasmus MC, University Medical Center Rotterdam, Rotterdam, The Netherlands; 4 Department of Internal Medicine-Geriatrics, Institute for Biomedicine of Aging (IBA), Friedrich-Alexander-Universität Erlangen-Nürnberg, Nürnberg, Germany; 5 Department of Geriatrics, Healthy Ageing Research Centre, Medical University of Lodz, Lodz, Poland; 6 Department of Physical Therapy, Recanati School for Community Health Professions at the faculty of Health Sciences, Ben-Gurion University of the Negev, Beer-sheva, Israel; 7 Maccabi Health Organization, Tel Aviv-Yafo, Israel; 8 Department of Medical Sciences, Uppsala University, Uppsala, Sweden; 9 Division of Family Medicine, Department of Neurobiology, Care Sciences and Society, Karolinska Institutet, Stockholm, Sweden; 10 School of Health and Social Studies, Dalarna University, Falun, Sweden; 11 Department of Internal Medicine, Medical University of Graz, Graz, Austria; 12 Geriatric Department, Hospital Clínico San Carlos, Martín Lagos S/N, 28040 Madrid, Spain; 13 Laboratory of Geriatric Pharmacoepidemiology and Biostatistics, IRCCS INRCA, Via S. Margherita 5, 60124 Ancona, Italy; 14 Italian National Research Center on Aging (IRCCS INRCA), Ancona, Fermo and Cosenza, Italy

**Keywords:** Older adults, Sarcopenia, Chronic kidney disease, Albuminuria, EWGSOP2, Estimated glomerular filtration rate

## Abstract

**Background:**

Loss of muscle mass and function may be more pronounced in older adults with chronic kidney disease (CKD) and with albuminuria. Thus, we investigated the prevalence of sarcopenia among community-dwelling older adults according to kidney function and grade of albuminuria. We also explored differences in the prevalence of sarcopenia according to three different equations for the estimation of glomerular filtration rate (eGFR).

**Methods:**

A cross-sectional analysis of 1420 community-dwelling older adults (≥75 years old) included in the SCOPE study, a multicenter prospective cohort study, was conducted. Comprehensive geriatric assessment including short physical performance battery (SPPB), handgrip strength test and bioelectrical impedance analysis (BIA) was performed. Sarcopenia was defined using the updated criteria of the European Working Group on Sarcopenia in Older People (EWGSOP2). eGFR was calculated using Berlin Initiative Study (BIS), Chronic Kidney Disease Epidemiological Collaboration (CKD-EPI) and Full Age Spectrum (FAS) equations, and urinary albumin-to-creatinine ratio (ACR) was collected to categorize CKD according to Kidney Disease Improving Global Outcomes guidelines.

**Results:**

Median age was 79.5 years (77.0–83.0), 804 (56.6%) were women. Using EWGSOP2 definition, 150 (10.6%) participants met diagnostic criteria for sarcopenia. Moreover, 85 (6%) participants had severe sarcopenia. Sarcopenia was more prevalent in participants with more advanced stages of CKD according to BIS eq. (9.6% in stages 1 and 2 and 13.9% in stages 3a, 3b and 4, *p* = 0.042), and also according to CKD-EPI (9.8% vs. 14.2%, p = 0.042) and FAS although not reaching statistical signification (9.8% vs. 12.7%, *p* = 0.119). Thus, differences in prevalence are observed among CKD categories as estimated by different equations. Prevalence of sarcopenia was also higher with increasing albuminuria categories: 9.3% in normoalbuminuric, 13.2% in microalbuminuric and 16.8% in macroalbuminuric participants, (*p* = 0.019).

**Conclusions:**

Sarcopenia is common among community-dwelling older adults, especially among those with more advanced CKD categories, with prevalence estimates differing slightly depending on the equation used for the estimation of eGFR; as well as among those with higher albuminuria categories.

## Background

The ageing process is characterised by quantitative and qualitative changes in body composition. Those affecting skeletal muscle mass and function are among the most relevant [1]. Sarcopenia is a muscle disease rooted in adverse muscle changes that accrue across a lifetime and is common among older adults, while it can also occur earlier in life. It represents a major cause of falls, is associated with other adverse health outcomes and predicts disability and mortality in older people [2]. Many definitions of sarcopenia have been proposed, even through various consensus from several societies such as the European Society of Clinical Nutrition (ESPEN) [3], European Working Group on Sarcopenia in Older People (EWGSOP) [4], International Working Group on Sarcopenia (IWGS) [5], Society on Sarcopenia, Cachexia and Wasting Disorders (SCWD) [6], Asian Working Group for Sarcopenia (AWGS) [7] and Foundation for the National Institutes of Health (FNIH) [8]. Differences exist in the criteria used for an operational definition, the tools used to measure them including different methods to adjust for body size, and the cut-off points used for each variable; thus leading to heterogeity in research studies and clinical practice, and to differences in prevalence estimates [9–11]. Recently, the EWGSOP updated the original definition of sarcopenia (EWGSOP2) [12], considering muscle strength as a central determinant of sarcopenia, suggesting specific tools and cut-off points for each variable defining sarcopenia, and also proposing a clinical algorithm for the identification, diagnosis and severity assessment of sarcopenia in a stepwise fashion. Thus, sarcopenia is considered probable when low muscle strength is detected, confirmed when low muscle mass is also evidenced, and severe when additionally low physical performance is present.

Chronic kidney disease (CKD) prevalence increases with age, and is often associated with additional comorbid conditions. In patients with CKD, loss of muscle mass is much more intense and the first signs of sarcopenia are observed in younger patients than it is expected [13]. Sarcopenia is more common among patients in the most advanced stages of CKD and is significantly associated with glomerular filtration rate (GFR) decline [14, 15]. Moreover, a bidirectional interaction between sarcopenia and albuminuria has been reported, sarcopenia is more prevalent in individuals with albuminuria than in those without; furthermore, increased albuminuria is independently associated with low muscle mass in patients with type 2 diabetes [16, 17].

The present study aimed to investigate the prevalence of sarcopenia as derived from the recently updated EWGSOP2 definition among a clinically relevant source of community-dwelling older adults, and to further assess its distribution according to different kidney function categories and grades of albuminuria, in the frame of the Screening for Chronic Kidney Disease among Older People across Europe (SCOPE) study. A second objective was to explore possible differences between three distinct equations for the estimation of GFR and the prevalence of sarcopenia.

## Methods

### Study design and participants

This cross-sectional study used data from the SCOPE study (European Grant Agreement no. 436849), a multicenter 2-year prospective cohort study involving patients older than 75 years attending geriatric and nephrology outpatient services in participating institutions in Austria, Germany, Israel, Italy, the Netherlands, Poland and Spain. Methods of the SCOPE study have been extensively described elsewhere [18]. Patients were requested to sign a written informed consent before entering the study. The study protocol was approved by ethics committees at all participating institutions, and complies with the Declaration of Helsinki and Good Clinical Practice Guidelines. Briefly, exclusion criteria were: a. Age < 75 years; b. End-stage renal disease (ESRD) (eGFR < 15 ml/min/1.73 m^2^) or dialysis at the time of enrollment; c. History of solid organ or bone marrow transplantation; d. Active malignancy within 24 months prior to screening or metastatic cancer; e. Life expectancy less than 6 months; f. Severe cognitive impairment (Mini Mental State Examination < 10); g. Any medical or other reason (e.g. known or suspected inability of the patient to comply with the protocol procedure) in the judgement of the investigators, that the patient is unsuitable for the study; h. Unwilling to provide consent and those who cannot be followed-up. After obtaining written informed consent, all participants underwent an extensive baseline visit including routine laboratory analysis and comprehensive geriatric assessment (CGA). The baseline visit was followed by follow-up visits at 12 and 24 months with intermediate phone contacts at 6 and 18 months. Only baseline data were used in the present study.

Overall, 2461 participants were intially enrolled in the study. Of them, 204 participants with missing serum creatinine and/or urinary albumin-to-creatinine ratio (ACR) were excluded, thus leaving a sample of 2257 participants to be included in the initial analysis. For the aim of the present study, only those participants in whom sarcopenia could be assessed in its three components (i.e., muscle strength, muscle mass and physical performance) were considered. Data for muscle strength, as assessed by grip strength; muscle mass, as assessed by bioelectrical impedance analysis (BIA); and physical performance, as assessed by the Short Physical Performance Battery (SPPB) were available for 2138 (94.7%), 1462 (64.8%) and 2256 (99.9%) participants respectively. Participants with missing data mainly included those physically unable or unsteady, those presenting arthralgia or arthritis, those with an implanted cardioverter-defibifrillator or pacemaker, or those not assessed due to any other safety reason in the judgement of the investigators. 1420 participants were finally included, for whom demographic and clinical characteristics were analysed. Anthropometric measures were collected and body mass index (BMI) was calculated as recommended in the ESPEN guidelines [19]. Cognitive function was assessed with the Mini Mental State Examination (MMSE) [20]; depressive symptoms were assessed with the Geriatric Depression Scale (GDS) in its short form [21]; the ability to perform activities of daily living (ADL) [22] and instrumental activities of daily living (IADL) [23] was also assessed. The Cumulative Illness Rating Scale for geriatrics (CIRS-G) [24] was administered to account for comorbidity burden. There were no statistically significant differences between both groups in age, gender, living alone rate, education years, ADL score, MMSE score, number of chronic medications and serum cretinine levels, although higher IADL score, GDS score and CIRS-G total score (though not higher severity index) were observed among the excluded study participants.

### Assessment of sarcopenia

Following the revised EWGSOP2 criteria for an operational definition of sarcopenia [12], all three components, i.e. muscle strength, muscle mass, and physical performance were assessed. Probable sarcopenia was identified when low muscle strength was present, diagnosis of sarcopenia was confirmed when low muscle strength and low muscle mass were both evidenced, and criteria for severe sarcopenia were met when sarcopenia concurred with low physical performance.

Muscle strength was assessed through the handgrip strength test [25], using a hydraulic grip strength dynamometer (Model J00105 JAMAR Hydraulic Hand, Lafayette Instrument Company, USA). Participants were encouraged to squeeze as hard as they could, 3 attempts were allowed for each hand alternating sides and the maximum measurement was registered. EWGSOP2 recommended cut-off points for low muscle strength were used, < 27 kg for men and < 16 kg for women.

Body composition in terms of fat and fat-free mass was assessed by BIA using the AKERN BIA 101 New Edition 50 kHz monofrequency device (AKERN SRL, Florence, Italy). Appendicular skeletal muscle mass (ASM) was estimated using the Sergi et al. equation [26], a cross-validated equation for standardisation specifically derived from older European populations, as recommended by the EWGSOP2 consensus. A decision was made to apply no adjustment for body size to ASM measures, as also contemplated in the consensus. Following the EWGSOP2 cut-off points, low muscle mass was defined by an ASM < 20.0 kg for men and < 15 kg for women.

Physical performance was assessed by the SPPB, a composite test consisting of a balance test (ability to stand for 10 s with feet close together side by side, then in semi-tandem and then in full-tandem positions), a gait speed assessment (usual time to walk 4 m), and a chair stand test (time to raise from a chair and return to the seated position 5 times without using arms) [27]. A score from 0 to 4 was assigned to each test, thus summing up to a maximum total score of 12. As suggested by the EWGSOP2 consensus, a total score of ≤8 was considered to indicate low physical performance.

### Assessment of kidney function

Serum creatinine was measured at local level by standard methods. Creatinine-based eGFR was calculated using the following equations:

Berlin Initiative Study (BIS) [28]:

$$ 3736\ \mathrm{x}\ {\mathrm{creatinine}}^{\hbox{-} 0.87}\mathrm{x}\ {\mathrm{age}}^{\hbox{-} 0.95}\mathrm{x}\ 0.82\left(\mathrm{if}\ \mathrm{female}\right) $$

Chronic Kidney Disease Epidemiological Collaboration (CKD-EPI) [29]:

 Female

$$ {\displaystyle \begin{array}{c}\left(\mathrm{Scr}\le 0.7\right)\kern1.75em \mathrm{eGFR}=144\ \mathrm{x}\ {\left(\mathrm{Scr}/0.7\right)}^{\hbox{-} 0.329}\mathrm{x}\ {(0.993)}^{\mathrm{Age}}\\ {}\left(\mathrm{Scr}>0.7\right)\kern1.75em \mathrm{eGFR}=144\ \mathrm{x}\ {\left(\mathrm{Scr}/0.7\right)}^{\hbox{-} 1.209}\mathrm{x}\ {(0.993)}^{\mathrm{Age}}\end{array}} $$

Male

$$ {\displaystyle \begin{array}{c}\left(\mathrm{Scr}\le 0.9\right)\kern0.75em \mathrm{eGFR}=141\ \mathrm{x}\ {\left(\mathrm{Scr}/0.9\right)}^{\hbox{-} 0.411}\mathrm{x}\ {(0.993)}^{\mathrm{Age}}\\ {}\left(\mathrm{Scr}>0.9\right)\kern0.75em \mathrm{eGFR}=141\ \mathrm{x}\ {\left(\mathrm{Scr}/0.9\right)}^{\hbox{-} 1.209}\mathrm{x}\ {(0.993)}^{\mathrm{Age}}\end{array}} $$

Full Age Spectrum (FAS) [30]:

$$ \left[107.3/\left(\mathrm{creatinine}/\mathrm{Q}\right)\right]\ \mathrm{x}\ 0{.988}^{\left(\mathrm{Age}\hbox{-} 40\right)}\mathrm{for}\ \mathrm{age}>40\ \mathrm{years} $$

Q = median Scr value for age−/sex-specific healthy populations.

Categories of CKD were defined according to Kidney Disease Improving Global Outcomes (KDIGO) guidelines [31], moreover CKD categories were we further combined into two groups, i.e., eGFR ≥60 ml/min/1.73 m^2^ (categories 1 and 2) and eGFR < 60 ml/min/1.73 m^2^ (categories 3a, 3b and 4). Albumin in urine was detected by urine spot analysis and expressed as mg albumin per gram urine (mg/g), and albumin-to-creatinine ratio (ACR) was calculated and reported as mg albumin per gram creatinine (mg/g). Categories of albuminuria were also defined according to KDIGO guidelines, thus, normoalbuminuria was defined as ACR < 30 mg/g, microalbuminuria as ACR 30–300 mg/g and macroalbuminuria as ACR > 300 mg/g.

### Statistical analysis

All variables were checked for normality by the Kolmogorov–Smirnov test. Continuous and normally variables are expressed as mean and standard deviation. Non-normally distributed variables are expressed as median and interquartile difference. Categorical variables are expressed as number and percentage.

The association between categorical variables was analyzed by the Chi-square test, with the correction of continuity when indicated. The relation between quantitative variables, according to sarcopenia categories, was performed by ANOVA test. Statistical significance was set at *p* < 0.05. All statistical analyses were performed with SPSS version 24 (SPSS Inc., Chicago, IL, USA) and MedCalc (JMP® statistics software, USA).

## Results

General characteristics of the 1420 participants evaluated are presented in Table 1. Median age was 79.5 years (77.0–83.0), there were 804 (56.6%) women and 337 participants (23.7%) were living alone. Regarding daily life activities, median ADL score was 0.0 (0.0–1.0), and 55 participants (3.9%) were considered as dependent; median IADL score was 2.0 (0.0–8.0), and 590 participants (41.5%) were considered as dependent. With respect to cognition, median MMSE score was 29.0 (27.0–30.0), with 104 participants (7.3%) showing MMSE scores < 24. Regarding depressive symptoms, median GDS score was 2.0 (1.0–4.0), and 170 participants (12%) exhibited scores of 5 and higher. The median CIRS-G comorbitidy total score was 7.0 (5.0–11.0), while median CIRS-G severity index was 1.5 (1.2–1.8). The median number of current medications was 6.0 (4.0–9.0).

**Table 1:** General characteristics of the study population

	*N* = 1420
Age, years	79.5 (77.0–83.0)
Women	804 (56.6%)
Living alone	337 (23.7%)
Education, years	11.0 (8.0–14.0)
ADL score	0.0 (0.0–1.0)
ADL dependent	55 (3.9%)
IADL score	2.0 (0.0–8.0)
IADL dependent	590 (41.5%)
MMSE score	29.0 (27.0–30.0)
MMSE < 24	104 (7.3%)
GDS score	2.0 (1.0–4.0)
GDS > 5	170 (12.0%)
CIRS-G total score	7.0 (5.0–11.0)
CIRS-G severity index	1.5 (1.2–1.8)
Number of current medications	6.0 (4.0–9.0)
Creatinine, mg/dl	0.93 (0.78–1.21)
BIS eGFR, ml/min/1.73 m^2^	56.4 (45.8–64.8)
≥ 90	9 (0.6%)
60–89	538 (37.9%)
45–59	542 (38.2%)
30–44	259 (18.2%)
15–29	72 (5.1%)
≥ 60	547 (38.5%)
< 60	873 (61.5%)
CKD-EPI eGFR, ml/min/1.73 m^2^	68.4 (52.6–80.7)
≥ 90	42 (3.0%)
60–89	884 (62.3%)
45–59	247 (17.4%)
30–44	163 (11.5%)
15–29	84 (5.9%)
≥ 60	926 (65.2%)
< 60	494 (34.8%)
FAS eGFR, ml/min/1.73 m^2^	56.8 (44.7–66.5)
≥ 90	26 (1.8%)
60–89	553 (38.9%)
45–59	480 (33.8%)
30–44	253 (17.8%)
15–29	108 (7.6%)
≥ 60	579 (40.8%)
< 60	841 (59.2%)
ACR, mg/g	12.6 (4.7–32.8)
< 30	1037 (73.0%)
30–300	288 (20.3%)
> 300	95 (6.7%)
Anthropometric measurements	
height, cm	162.0 (156.0–170.0)
weight, kg	72.0 (62.7–82.0)
BMI, kg/m^2^	27.0 (24.4–30.0)
BIA parameters	
FFM, percentage	69.0 (62.9–75.0)
FFM, kg	48.5 (42.6–57.5)
FFMI, kg/m^2^	18.5 (17.1–20.2)
FM, percentage	31.0 (24.9–37.1)
FM, kg	21.9 (16.7–28.6)
FMI, kg/m^2^	8.3 (6.3–10.9)
ASM, percentage	0.25 (0.23–0.28)
ASM, kg	18.1 (15.2–21.7)
ASMI, kg/m^2^	6.9 (6.2–7.6)
Grip strength, kg	24.0 (18.0–31.0)
SPPB score	10.0 (8.0–11.0)

Note: continuous variables are expressed as median and interquartile difference. *Abbreviations* *ACR* Albumin-to-creatinine ratio, *ADL* Activities of daily living, *ASM* Appendicular skeletal muscle mass, *ASMI* Appendicular skeletal muscle mass index, *BIA* Bioelectrical impedance analysis, *BIS* Berlin Initiative Study, *BMI* Body mass index, *CIRS-G* Cumulative illness rating scale for geriatrics, *CKD-EPI* Chronic Kidney Disease Epidemiological Collaboration, *eGFR* Estimated glomerular filtration rate, *FAS* Full Age Spectrum, *FFM* Fat-free mass, *FFMI* Fat-free mass index, *FM* Fat mass, *FMI* Fat mass index, *GDS* Geriatric depression scale, *IADL* Instrumental activities of daily living, *MMSE* Mini mental state examination, *SSPB* Short physical performance battery

Median BMI was 27.0 kg/m^2^ (24.4–30.0) and according to a BMI cut-off value of ≥30 kg/m^2^, 359 participants (25.3%) were obese, 143 men (23.2%) and 216 women (26.9%); whereas according to fat mass percentage (FM%) cut-off values of ≥25% for men and ≥ 35% for women, 768 participants (54.1%) were obese, 355 men (57.6%) and 413 women (51.4%), median FM% was 31.0 (24.9–37.1).

### Kidney function and albuminuria

The median serum creatinine concentration was 0.93 mg/dl (0.78–1.21) (Table 1). Median eGFR values (ml/min/1.73 m^2^) according to three different equations were 56.4 (45.8–64.8) (BIS), 68.4 (52.6–80.7) (CKD-EPI) and 56.8 (44.7–66.5) (FAS). Distribution of participants among eGFR categories and according to the different equations is presented in Table 1. When considered together, 873 participants (61.5%) according to BIS, 494 (34.8%) according to CKD-EPI, and 841 (59.2%) according to FAS had stage 3a, 3b or 4 CKD (eGFR < 60 ml/min/1.73 m^2^). The median ACR value was 12.6 mg/g (4.7–32.8), and 1037 participants (73%) had normoalbuminuria, 288 (20.3%) had microalbuminuria and 95 (6.7%) had macroalbuminuria.

### Sarcopenia components and categories (Table 2)

Median grip strength was 24.0 kg (18.0–31.0), and 306 (21.5%) participants showed low muscle strength according to the aforementioned cut-off values, 136 men (22.1%) and 170 women (21.1%) (*p* = 0.672). Median ASM as derived from BIA was 18.1 kg (15.2–21.7), and following the EWGSOP2 recommended cut-off values, 463 participants (32.6%) exhibited low muscle mass, with a statistically significant female predominance: 312 women (38.8%) vs. 151 men (24.5%) (*p* < 0.001). Regarding SPPB, median score was 10.0 (8.0–11.0), and considering the recommended cut-off value of ≤8 points, 440 participants (31%) had low physical performance, a condition also exhibited more often by female participants (*p* = 0.003): 275 women (34.2%) vs. 165 men (26.8%).

**Table 2:** Summary of sarcopenia components and sarcopenia categories, stratified by gender

	Total (*n* = 1420)	Men (*n* = 616)	Women (*n* = 804)	*p*-value
**Sarcopenia components**				
Low muscle strength	306 (21.5%)	136 (22.1%)	170 (21.1%)	0.672
Low muscle mass	463 (32.6%)	151 (24.5%)	312 (38.8%)	< 0.001
Low physical performance	440 (31.0%)	165 (26.8%)	275 (34.2%)	0.003
**Sarcopenia categories**				
No sarcopenia	1114 (78.5%)	480 (77.9%)	634 (78.9%)	0.672
Probable sarcopenia	156 (11.0%)	77 (12.5%)	79 (9.8%)	0.110
Confirmed sarcopenia	65 (4.6%)	26 (4.2%)	39 (4.9%)	0.573
Severe sarcopenia	85 (6.0%)	33 (5.4%)	52 (6.5%)	0.382

Nearly half of the study sample (599 participants, 42.2%) showed no derangement in either muscle strength, muscle mass or physical performance, a condition observed in 303 men (49.2%) and 296 women (26.8%). Prevalence of sarcopenia in the present study was 10.6%, with a total of 150 participants meeting criteria for sarcopenia, 59 men (9.6%) and 91 women (11.3%) (*p* = 0.29). Among the non-sarcopenic participants, 1114 (78.5% of the study sample) had normal muscle strength, 480 men (77.9%) and 634 women (78.9%); and 156 participants (11%) had low muscle strength, 77 men (12.5%) and 79 women (9.8%). Among the sarcopenic participants, 65 (4.6% of the study sample) had normal physical performance, 26 men (4.2%) and 39 women (4.9%); and 85 participants (6%) had low physical performance, 33 men (5.4%) and 52 women (6.5%), therefore meeting criteria for severe sarcopenia. Differences in rates of sarcopenia categories between men and women showed no statistical significance.

### Sarcopenia according to kidney function (Tables 3, 4, 5 and 6)

According to BIS (Table 3), sarcopenia was significantly more prevalent in the more advanced stages of CKD: 9.6% in stages 1 and 2 (eGFR ≥60 ml/min/1.73 m^2^) vs. 13.9% in stages 3a, 3b and 4 (eGFR < 60 ml/min/1.73 m^2^) (*p* = 0.024). Similarly, rates of severe sarcopenia were also higher in participants in CKD stages 3–4 than in CKD stages 1–2 (4.7% vs. 10.3%), with statistical significance (*p* = 0.005), and were highest in CKD stage 4 (13.9%).

**Table 3:** Prevalence of sarcopenia and sarcopenia categories according to BIS eGFR (ml/min/1.73m^2^) categories

	Non-sarcopenic (*n* = 1270)		Sarcopenic (*n* = 150)		*p*-value
BIS eGFR category	No sarcopenia (*n* = 1114)	Probable sarcopenia (*n* = 156)	Confirmed sarcopenia (*n* = 65)	Severe sarcopenia (*n* = 85)	
**≥90 (*n*** **= 9)**	6 (66.7%)		3 (33.3%)		0.026
	3 (33.3%)	3 (33.3%)	2 (22.2%)	1 (11.1%)	
**60–89 (*n*** **= 538)**	483 (89.8%)		55 (10.2%)		0.745
	440 (81.8%)	43 (8.0%)	26 (4.8%)	29 (5.4%)	
**45–59 (*n*** **= 542)**	496 (91.5%)		46 (8.5%)		0.046
	436 (80.4%)	60 (11.1%)	25 (4.6%)	21 (3.9%)	
**30–44 (*n*** **= 259)**	225 (86.9%)		34 (13.1%)		0.138
	188 (72.6%)	37 (14.3%)	10 (3.9%)	24 (9.3%)	
**15–29 (*n*** **= 72)**	60 (83.3%)		12 (16.7%)		0.084
	47 (65.3%)	13 (18.1%)	2 (2.8%)	10 (13.9%)	
**≥60 (*n*** **= 1089)**	985 (90.4%)		104 (9.6%)		0.024
	879 (80.7%)	106 (9.7%)	53 (4.9%)	51 (4.7%)	
**< 60 (*n*** **= 331)**	285 (86.1%)		46 (13.9%)		
	235 (71.0%)	50 (15.1%)	12 (3.6%)	34 (10.3%)	

*BIS* Berlin Initiative Study, *eGFR* Estimated glomerular filtration rate

**Table 4:** Prevalence of sarcopenia and sarcopenia categories according to CKD-EPI eGFR (ml/min/1.73m^2^) categories

	Non-sarcopenic (*n* = 1270)		Sarcopenic (*n* = 150)		*p*-value
CKD-EPI eGFR category	No sarcopenia (*n* = 1114)	Probable sarcopenia (*n* = 156)	Confirmed sarcopenia (*n* = 65)	Severe sarcopenia (*n* = 85)	
**≥90 (*n*** **= 42)**	37 (88.1%)		5 (11.9%)		0.774
	29 (69.0%)	8 (19.0%)	3 (7.1%)	2 (4.8%)	
**60–89 (*n*** **= 884)**	795 (89.9%)		89 (10.1%)		0.435
	720 (81.4%)	75 (8.5%)	46 (5.2%)	43 (4.9%)	
**45–59 (*n*** **= 247)**	226 (91.5%)		21 (8.5%)		0.246
	194 (78.5%)	32 (13.0%)	5 (2.0%)	16 (6.5%)	
**30–44 (*n*** **= 163)**	139 (85.3%)		24 (14.7%)		0.066
	112 (68.7%)	27 (16.6%)	9 (5.5%)	15 (9.2%)	
**15–29 (*n*** **= 84)**	73 (86.9%)		11 (13.1%)		0.436
	59 (70.2%)	14 (16.7%)	2 (2.4%)	9 (10.7%)	
**≥60 (*n*** **= 1173)**	1058 (90.2%)		115 (9.8%)		0.042
	943 (80.4%)	115 (9.8%)	54 (4.6%)	61 (5.2%)	
**< 60 (*n*** **= 247)**	212 (85.8%)		35 (14.2%)		
	171 (69.2%)	41 (16.6%)	11 (4.5%)	24 (9.7%)	

*CKD-EPI* Chronic Kidney Disease Epidemiological Collaboration, *eGFR* Estimated glomerular filtration rate

**Table 5:** Prevalence of sarcopenia and sarcopenia categories according to FAS eGFR (ml/min/1.73m^2^) categories

	Non-sarcopenic (*n* = 1270)		Sarcopenic (*n* = 150)		*p*-value
FAS eGFR category	No sarcopenia (*n* = 1114)	Probable sarcopenia (*n* = 156)	Confirmed sarcopenia (*n* = 65)	Severe sarcopenia (*n* = 85)	
**≥90 (*n*** **= 26)**	22 (84.6%)		4 (15.4%)		0.420
	16 (61.5%)	6 (23.1%)	2 (7.7%)	2 (7.7%)	
**60–89 (*n*** **= 553)**	496 (89.7%)		57 (10.3%)		0.802
	456 (82.5%)	40 (7.2%)	29 (5.2%)	28 (5.1%)	
**45–59 (*n*** **= 480)**	437 (91.0%)		43 (9.0%)		0.160
	380 (79.2%)	57 (11.9%)	22 (4.6%)	21 (4.4%)	
**30–44 (*n*** **= 253)**	225 (89.0%)		28 (11.1%)		0.774
	188 (74.3%)	37 (14.6%)	9 (3.6%)	19 (7.5%)	
**15–29 (*n*** **= 108)**	90 (83.3%)		18 (16.7%)		0.032
	74 (68.5%)	16 (14.8%)	3 (2.8%)	15 (13.9%)	
**≥60 (*n*** **= 1059)**	955 (90.2%)		104 (9.8%)		0.119
	852 (80.5%)	103 (9.7%)	53 (5.0%)	51 (4.8%)	
**< 60 (*n*** **= 361)**	315 (87.3%)		46 (12.7%)		
	262 (72.6%)	53 (14.7%)	12 (3.3%)	34 (9.4%)	

*eGFR* Estimated glomerular filtration rate, *FAS* Full Age Spectrum

**Table 6:** Prevalence of sarcopenia and sarcopenia categories according to ACR (mg/g) categories

	Non-1sarcopenic (*n* = 1270)		Sarcopenic (*n* = 150)		*p*-value
ACR category	No sarcopenia (*n* = 1114)	Probable sarcopenia (*n* = 156)	Confirmed sarcopenia (*n* = 65)	Severe sarcopenia (*n* = 85)	
**< 30 (*n*** **= 1037)**	941 (90.7%)		96 (9.3%)		0.008
	842 (81.2%)	99 (9.5%)	46 (4.4%)	50 (4.8%)	
**30–300 (*n*** **= 288)**	250 (86.8%)		38 (13.2%)		0.104
	211 (73.3%)	39 (13.5%)	14 (4.9%)	24 (8.3%)	
**> 300 (*n*** **= 95)**	79 (83.2%)		16 (16.8%)		0.039
	61 (64.2%)	18 (18.9%)	5 (5.3%)	11 (11.6%)	

*ACR* Albumin-to-creatinine ratio

Sarcopenia prevalence was also higher in CKD stages 3–4 compared with CKD stages 1–2 (*p* = 0.042) when eGFR was estimated using CKD-EPI equation (Table 4), although prevalence rates differed slightly: 9.8% vs. 14.2%. Rates of severe sarcopenia were 5.2% vs. 9.7% respectively, though difference was not statistically significant (*p* = 0.105), with rates being lowest in CKD stage 1 (4.8%), and highest in CKD stage 4 (10.7%).

Finally, when eGFR was calculated with FAS formula (Table 5), prevalence of sarcopenia was 9.8% (eGFR ≥60 ml/min/1.73 m^2^) vs. 12.7% (eGFR < 60), though differences were not statistically significant (*p* = 0.119). Higher percentages of severely sarcopenic participants were observed among CKD stages 3–4 (9.4%) compared with stages 1–2 (4.8%) (p = 0.005), and were highest among CKD stage 4 participants (13.9%).

Therefore, higher rates of sarcopenia and severe sarcopenia were observed among participants in more advanced stages of CKD, irrespective of the equation used for the estimation of eGFR, although sarcopenia prevalence varied slightly in those with eGFR < 60 ml/min/1.73m^2^: 13.9% with BIS, 14.2% with CKD-EPI and 12.7% with FAS equations.

Finally, the distribution of participants according to ACR categories also yielded significantly higher prevalence rates of sarcopenia with increasing albuminuria categories: 9.3% in normoalbuminuria, 13.2% in microalbuminuria and 16.8% in macroalbuminuria (*p* = 0.019). Similarly, as ACR rised, higher rates of severe sarcopenia were observed: 4.8, 8.3 and 11.6% respectively, although without statistical significance (*p* = 0.297).

## Discussion

In the SCOPE study, where community-dwelling older adults (75 years and older) from 7 European countries within a wide range of kidney function were evaluated (from normal to stage 4 CKD, therefore excluding patients with ESRD); one of ten community-dwelling older adults had sarcopenia, and 6% of the evaluated participants had severe sarcopenia. Women had higher rates of low muscle mass and more often exhibited low physical performance than men; and although sarcopenia was present in 11.3% of women and 9.6% of men, and severe sarcopenia in 6.5% of women and 5.4% of men, differences were not statistically significant. Our study found that a higher prevalence of sarcopenia is observed among participants with poorer kidney function categories (CKD stages 3–4) compared with participants with better kidney function categories (CKD stages 1–2), irrespective of the equation used to estimate eGFR (BIS, CKD-EPI or FAS), with prevalence rates of 13.9, 14.2 and 12.7% respectively, according to the EWGSOP2 revised criteria for sarcopenia [12]. These findings are clinically relevant, especially when considering that sarcopenia may affect mobility and increase the risk of falls among older individuals. Indeed, muscle mass and strength were formerly found to be linearly associated with mobility impairment [32], and the contribution of sarcopenia to unexplained falls is still to be elucidated [33]. Thus, our results suggest that the assessment of sarcopenia may be helpful in identifying older CKD patients at risk of falling and implementing inherent preventive measures.

Previous studies have found differences in prevalence estimates of sarcopenia when operational criteria proposed from various existing consensus were compared [9–11]. A recent systematic review and meta-analysis [10] found higher prevalence rates when a single measure of muscle mass was used to define sarcopenia instead of composite definitions, and that prevalence estimates also depended on the use of BIA vs. dual-energy X-ray absorptiometry (DXA) to assess muscle mass, the cut-off points employed or even the method of adjustment for body size. Recently, EWGSOP2 criteria have been found to yield lower prevalence estimates (9.3% overall, 11.9% in men and 6.7% in women) than EWGSOP criteria (20.8% overall, 25.5% in men and 16.2% in women) [11]. The combination of different tools and adjustments also impacted prevalence rates; specifically, the use of grip strength and ASM to define sarcopenia yielded a prevalence of 11.7%, resembling that observed in the SCOPE study though differing on the gender predominance; likewise, the use of grip strength, ASM and SPPB to define severe sarcopenia yielded a prevalence of 2.2%. Other recent studies in community-dwelling older adults have used EWGSOP2 criteria [34, 35] with varying prevalence rates of sarcopenia (20 and 3.4% respectively) and severe sarcopenia (1.8% vs. 3.2%), though different assessment methods were employed.

Sarcopenia has been reported to be common in community-dwelling adults with CKD, with prevalence rising markedly with declining kidney function [36]. Specifically, in adults with eGFR ≥90, eGFR 60–89 and eGFR < 60 ml/min/1.73m^2^ prevalence was 26.6, 38.9, and 60.1% respectively. It is worth noting that only muscle mass with a less restringing cut-off was employed to define sarcopenia, which could account for the higher prevalence observed as compared with our results. Sarcopenia has been found to be highly prevalent among patients with CKD [37], and more prevalent among persons with lower eGFR, with stage 4 CKD being independently associated with an increased likelihood of sarcopenia. CKD has been reported to be a major risk factor of sarcopenia in community-dwelling older men [38], and that even stage 3 CKD had a more than threefold risk of skeletal muscle mass (SMM) reduction.

In patients with stages 3–5 CKD on conservative therapy, prevalence of sarcopenia was found to vary according to the method used to assess muscle mass, with BIA derived SMMI yielding lower estimates (5.9%) as opposed to the use of midarm muscle circumference (9.8%) or subjective global assessment (9.4%) as surrogates of muscle mass [39]. Noteworthy, this study assessed both muscle mass and strength components of sarcopenia, which could account for the lower prevalence rates observed, far more similar to our results. Another study in a Korean population [40] found that prevalence of sarcopenia was higher in patients even with early stage kidney disease, with prevalence raising as the stage of CKD increased from eGFR ≥90, to eGFR 60–89.9, and eGFR < 60 ml/min/1.73m^2^ (2.6, 5.6 and 18.1% respectively in men, and 5.3, 7.1 and 12.6% respectively in women), although a statistically significant association between sarcopenia and stage of CKD was found in men but not in women. In this regard, our study found a higher prevalence among women across virtually all CKD stages. Other studies in CKD patients have observed variable prevalence rates of sarcopenia: 34.5% (stages 2-3a) and 65.5% (stages 3b-5) [14]; 37% (stages 3–5) [41]; 14% (stages 3–5) greater in men (16%) than in women (8%) and with a significant positive relationship between ASM and GFR [15]; and 12.5% (stages 3b-4 in men aged 60–74) or 55% (stages 3b-4 in men aged > 75) [42].

Although several previous studies have evaluated the relationship between muscle mass and/or muscle strength and kidney function, to our knowledge this is the first study to assess sarcopenia according to CKD stages incorporating the revised EWGSOP2 criteria for its definition. Furthermore, varying methods for estimating GFR have been employed, mainly the CKD-EPI and Modification of Diet in Renal Disease (MDRD) equations, whereas few studies have employed measured GFR, e.g., through iohexol clearance method [15]. Sarcopenia is a muscle disease closely related to ageing, and its prevalence increases sharply within ageing populations, in which concern exists about the accuracy of GFR estimating equations. Performance of such equations has been compared against equations specifically developed in older populations with varying results [43]. In the SCOPE study it has been demonstrated that CKD-EPI, BIS and FAS equations cannot be considered interchangeable in a community-dwelling older population, and that muscle mass may represent a major source of discrepancy among equations [data not published]. In the present study, the percentage of individuals with sarcopenia in CKD stages 3–4 varied slightly according to the equations employed: 13.9% for BIS, 14.2% for CKD-EPI and 12.7% for FAS equation.

An increased risk of albuminuria, which is associated with mortality, cardiovascular disease and CKD progression, has been reported in patients with sarcopenia and vice versa, independently of CKD [17]. This association has been found to be particularly strong in the older adult population [44] and synergistic with obesity, and also demonstrated in patients with type 2 diabetes [16]. Furthermore, sarcopenia has been associated with an increased risk of progression of albuminuria in a retrospective study of diabetic patients [45], and with an increased risk of incident albuminuria in a recent prospective study in participants without CKD [46]. Common underlying mechanisms have been suggested to account for this association, such as inflammation, insulin resistance, endothelial dysfunction and renin-angiotensin-aldosterone system activation. In the present study a higher prevalence of sarcopenia is observed with increasing ACR category: 9.3% in normoalbuminuric participants, 13.2% in the microalbuminuric group and 16.8% in participants with macroalbuminuria (*p* = 0.019).

Some strengths of the present study are inherent to the SCOPE study design, which included a remarkable number of community-dwelling older adults from different centers across Europe, following highly inclusive criteria to ensure the enrolment of a representative sample of real-world outpatients, within a wide range of kidney function categories. Though previous studies have investigated sarcopenia prevalence, few studies have taken CKD status into account, especially regarding CKD patients not on dialysis. Furthermore, the association of higher rates of sarcopenia and kidney disease may depend not only on CKD stage or degree of eGFR but also on albuminuria status, with fewer studies assessing both variables concomitantly or comparing different eGFR equations. Moreover, as new revised definitions of sarcopenia arise following the increasing knowledge of this condition, the prevalence rates, the association with risk factors or predisposing conditions, and even the association with outcomes may differ from what was previously assumed. Thus, this is the first study to investigate sarcopenia in patients not in ESRD incorporating the newly recommended criteria by the EWGSOP2 consensus for the definition of sarcopenia and its subtypes through its three components (i.e.: muscle mass, muscle strength and physical performance); and also assessing not only different equations for the estimation of GFR, but also albuminuria status as relevant measures related to kidney function.

Some limitations deserve consideration. First, the present study represents a cross-sectional analysis of SCOPE study, and causality in the relationship between sarcopenia and kidney function or albuminuria cannot be established. Second, the exclusion of ESRD participants from the SCOPE study limits its applicability in this group of patients. Third, although participants not included in the analysis because of missing data on any of the sarcopenia components did not seem to differ on the majority of general characteristics from those who completed sarcopenia assessment; a poorer clinical status, poorer comorbidity profile or higher physical disability in those participants could have led to an underestimation of sarcopenia prevalence in our study. Fourth, the use of BIA for the estimation of ASM, although endorsed by different consensuses because of its affordability, availability and portability, may exhibit some limitations as compared with DXA measurements or with magnetic resonance imaging/computed tomography (currently considered as gold standards for non-invasive assessment of muscle mass); and hydration status has to be taken into account as it may influence results, especially in CKD patients. Moreover, concern exists regarding the use of height-adjusted ASM to correct for body size, as it tends to underestimate the prevalence of sarcopenia and may not be suitable for all populations. As EWGSOP2 consensus makes no recommendation to adjust for body size, a decision was made to employ non-adjusted ASM as a measure of muscle mass. Finally the use of non-creatinine based equations for the estimation of GFR, such as eGFR based on cystatin C, might be more accurate for the evaluation of sarcopenia in these patients; thus studies comparing eGFR equations based on cystatin C or incorporating measured GFR by iohexol clearance may be of paramount relevance. Creatinine is a metabolic product of creatine and phosphocreatine arising from the muscle compartment, which is directly related to muscle mass. Thus, muscle wasting may lead to an overestimation of eGFR in sarcopenic patients, which should be taken into account when evaluating such patients. Further research is needed to investigate the underlying mechanisms of sarcopenia among older adults with CKD and albuminuria.

## Conclusions

The main finding from our study is the relevant prevalence of sarcopenia observed among community-dwelling European adults aged 75 years and older, using the most recent diagnostic criteria for sarcopenia endorsed by the EWGSOP2 consensus. Participants within poorer eGFR categories, irrespective of the equation used for its calculation, have a higher prevalence of sarcopenia and are more often severely sarcopenic. There are though some differences in prevalence according to the eGFR formula used. Moreover, participants within higher albuminuria categories are more often sarcopenic, with higher rates of severe sarcopenia. Therefore, prompt assessment of sarcopenia status may be warranted in the usual care of older people with impaired kidney function and/or albuminuria, which could allow for its early detection and trigger proper interventions.

## Data Availability

Data will be available for SCOPE researchers through the project website (www.scopeproject.eu).

## Abbreviations

ACR: Albumin-to-creatinine ratio
ADL: Activities of daily living
ASM: Appendicular skeletal muscle mass
ASMI: Appendicular skeletal muscle mass index
AWGS: Asian Working Group for Sarcopenia
BIA: Bioelectrical impedance analysis
BIS: Berlin Initiative Study
BMI: Body mass index
CGA: Comprehensive geriatric assessment
CIRS-G: Cumulative illness rating scale for geriatrics
CKD: Chronic kidney disease
CKD-EPI: Chronic Kidney Disease Epidemiological Collaboration
DXA: Dual-energy X-ray absorptiometry
eGFR: Estimated glomerular filtration rate
ESPEN: European Society of Clinical Nutrition
ESRD: End-stage renal disease
EWGSOP: European Working Group on Sarcopenia in Older People
FAS: Full Age Spectrum
FFM: Fat-free mass
FFMI: Fat-free mass index
FM: Fat mass
FMI: Fat mass index
FNIH: Foundation for the National Institutes of Health
GDS: Geriatric depression scale
GFR: Glomerular filtration rate
IADL: Instrumental activities of daily living
IWGS: International Working Group on Sarcopenia
KDIGO: Kidney Disease Improving Global Outcomes
MDRD: Modification of Diet in Renal Disease
MMSE: Mini mental state examination
SCOPE: Screening for Chronic Kidney Disease among Older People across Europe
SCWD: Society on Sarcopenia, Cachexia and Wasting Disorders
SMM: Skeletal muscle mass
SMMI: Skeletal muscle mass index
SSPB: Short physical performance battery
